# Developing context-specific competencies for epidemic and pandemic preparedness in the MENA region: a training needs assessment and Delphi approach

**DOI:** 10.3389/fpubh.2026.1778190

**Published:** 2026-04-20

**Authors:** Sarah Ibrahim, Rim Alaeddine, Zahraa Chamseddine, Nour Zaouk, Zeinab Awad, Zahi Abdul-Sater, Shadi Saleh

**Affiliations:** 1 Global Health Institute, American University of Beirut, Beirut, Lebanon; 2 Faculty of Health Sciences, American University of Beirut (AUB), Beirut, Lebanon

**Keywords:** context-specific competencies, Delphi method, epidemic preparedness, health systems strengthening, Middle East and North Africa (MENA), pandemic preparedness, public health workforce, training needs assessment

## Abstract

**Introduction:**

Epidemics and pandemics continue to expose persistent preparedness gaps across the Middle East and North Africa (MENA), particularly in fragile and conflict-affected settings. Strengthening workforce capacities through context-specific competencies is critical for improving preparedness and enhancing health system resilience.

**Methods:**

Guided by the European Centre for Disease Prevention and Control (ECDC) preparedness framework, the study employed a two-phase mixed-methods design comprising a Training Needs Assessment (TNA) and Delphi survey. The TNA was administered to 95 professionals from 18 MENA countries. Gaps between perceived importance and ability to perform were analyzed quantitatively alongside thematic analysis of open responses. To ensure expert validation of these competencies, the Delphi survey was conducted and included 10 regional experts involved in two iterative online rounds and a consensus meeting to refine and prioritize competencies.

**Results:**

The TNA identified significant training gaps across all five ECDC preparedness domains, with the highest needs observed in Policy Development, Adaptation, and Implementation, Detection and Assessment and Health Services. Qualitative findings revealed additional barriers, including resource shortages, weak governance, and limited access to training opportunities. The Delphi process refined these findings, allowing experts to prioritize competencies as basic, core, essential, or omitted. Of the 99 competencies, 71 were classified as essential, 23 as core, and 5 as basic. The results of the surveys directly informed the focus and content of the certificate in epidemic and pandemic preparedness.

**Conclusion:**

This study utilized the ECDC preparedness framework to assess and refine competencies for epidemic and pandemic preparedness in the MENA region. Through a TNA and Delphi approach, it produced validated, context-specific competencies that informed the design of a contextualized regional certificate. The findings underscored the critical need for targeted training programs that address both immediate and long-term challenges in health system resilience in the MENA region.

## 1 Introduction

Epidemics and pandemics continue to pose significant challenges to global health systems, exposing systemic vulnerabilities in preparedness and response capacities (1). The 21st century has witnessed a surge in infectious threats, including SARS-CoV-1, the 2013–2016 Ebola epidemic, and most recently, the global disruptions of COVID-19 and Mpox (2). While these crises have tested public health infrastructures worldwide, the Middle East and North Africa (MENA) region remains uniquely precarious. This vulnerability is driven by a series of compounding crises, where political instability and protracted conflicts intersect with high population mobility and fragmented health governance (3). Such a context necessitates preparedness efforts that are not only sustained but fundamentally grounded in regional realities rather than generic global models.

Countries across the region exhibit relatively low epidemic preparedness, characterized by limited capacities in early detection, assessment, surveillance, timely outbreak reporting, and effective public health response (4). These weaknesses have also been reflected in limited early warning and response capacity, inadequate integration of local scientific evidence into policymaking, and weak regional coordination, all of which have constrained timely and contextually appropriate outbreak control (4).

Despite the recurring threats, the MENA region lacks a systematically trained public health workforce equipped with context-specific competencies to effectively prepare for and respond to epidemics and pandemics (5). Existing capacity-building initiatives are not sufficiently adapted to the needs of the region and are not driven by social and economic realities. As a result, training priorities often fail to align with the actual operational needs faced by professionals in the region. While global competency frameworks—such as those established by the WHO or CDC—offer essential universal standards, they often fail to account for the operational constraints of resource-limited or conflict-affected settings. In addition, previous epidemics such as MERS-CoV and COVID-19 have underscored the importance of training and capacity building, yet preparedness efforts have largely been reactive rather than proactive where lessons from one outbreak are not institutionalized into policy or training systems, resulting in repeated vulnerabilities (6). For example, workforce assessments in Yemen and Syria revealed that fewer than one-third of healthcare workers had received formal outbreak preparedness training (7). This was reflected in the limited implementation and enforcement of proactive prevention and control measures, including inconsistent screening protocols at national borders. This pattern underscores the need for a systematic, adaptable framework that is evidence-based to support proactive and sustained health-system resilience in the region.

Preparedness across the MENA also reflects deep structural and contextual differences. While high-income countries such as Saudi Arabia and the United Arab Emirates showed stronger readiness through higher vaccination coverage and better system capacity (8, 9) conflict-affected countries like Yemen, Libya, and Syria faced critical shortages of trained staff, functional facilities, and essential supplies (10, 11). This demonstrates broader divides in resources, capacity, and governance, leaving fragile health systems at a disadvantage. Ongoing conflicts, economic strain, and large-scale displacement continue to widen these disparities (12, 13).

Building the competencies of the public health workforce is essential (14, 15) and must extend beyond traditional health workers to include professionals from other sectors to address workforce shortages and improve response. While previous studies and global frameworks have addressed preparedness broadly, limited research has systematically identified and validated context-specific preparedness competencies for the MENA region. To address this gap, an evidence-based consensus process designed to bridge the gap between perceived professional needs and regional operational realities. This study addresses this critical gap by developing a validated framework designed to move regional capacity-building beyond ad-hoc training sessions toward a professionalized, sustainable model. By identifying the specific skill sets required to navigate the MENA’s unique challenges, this study aims to: (a) analyze the functional gaps between the existing skills and the perceived importance of preparedness tasks among regional practitioners; (b) validate and prioritize a set of core competencies that reflect regional relevance and applicability, and (c) provide the evidence base for a contextualized professional certification designed to strengthen the long-term resilience of the regional health workforce.

## 2 Methods

To identify knowledge gaps and training needs among professionals in epidemic and pandemic preparedness in the MENA region, this study employed a sequential mixed-methods design integrating a quantitative-qualitative Training Needs Assessment (TNA) followed by a quantitative Delphi survey.

### 2.1 Training needs assessment

A training needs assessment survey was conducted to evaluate the training needs, barriers, and priorities related to epidemic and pandemic preparedness among key stakeholders across the MENA region. The survey structure was informed by the European Centre for Disease Prevention and Control (ECDC) framework for public health emergency preparedness, which provides a structured approach to strengthening preparedness for public health emergencies. This framework outlines core competencies, capacities, and strategic priorities essential for effective preparedness and response to infectious disease outbreaks and other public health threats. Specifically, the ECDC framework categorizes key epidemic and pandemic preparedness competencies into five main domains: Detection and Assessment, Policy Development, Adaptation, and Implementation, Health Services, Coordination and Communication, and Emergency Risk Communication (16). The framework provides a competency-based structure that aligns with the study’s aim of assessing individual preparedness capacities, rather than broader national-level capacities. Its focus on practical tasks relevant to frontline and public health professionals, along with its clear operational domains, made it transferable into measurable survey items (see Supplementary material 1 for full competency list used in the study).

To systematically identify gaps between current and required capacities among professionals working in epidemic and pandemic preparedness, a mixed-method approach was adopted using the Hennessy-Hicks (HH) training needs assessment tool supplemented by open-ended questions. The HH tool facilitates a comparative analysis of existing competencies and performance and perceived importance to enhance preparedness efforts (17). Participants rated competencies related to pandemic preparedness on a scale from 1 to 7 across two dimensions: their importance to the job (Score A) and the ability to perform each task (Score B).

Ratings covered the five ECDC domains: detection and assessment, coordination and communication, emergency risk communication, health services, and policy development, implementation and adaptation. The open-ended questions explored factors contributing to the lack of preparedness in the region, barriers to attending training programs, and the training needs of participants.

Participants were identified through a combination of desk review and snowball sampling, ensuring a diverse representation of stakeholders. The target population included public health officials, public health workers, government officials, public health emergency response managers, risk communicators, clinicians, and civil society leaders. The survey was administered online using LimeSurvey, and data collection was conducted remotely. It included sections on demographics, training needs, previous training experiences, and perceived barriers to epidemic and pandemic preparedness in the MENA region. The collected data was exported to Microsoft Excel for cleaning and further analysis. The online TNA survey is provided in the Supplementary material 2.

### 2.2 Delphi survey

To complement the TNA and establish expert consensus on priority competencies for a tailored certificate in epidemic and pandemic preparedness, a multistage Delphi survey was conducted. While the TNA identified gaps between the perceived importance of preparedness competencies and professionals’ ability to perform them across the region, the Delphi process engaged regional experts to review and prioritize competencies relevant to epidemic and pandemic preparedness. This structured, iterative process facilitated expert agreement on the essential competencies required for effective preparedness and response in the MENA region. The Delphi process followed three phases:

1. Competency Identification: a preliminary set of competencies was derived from the ECDC framework on public health emergency preparedness then reviewed for their relevance and potential inclusion in the certificate curriculum.
2. Two-Round Modified Delphi Survey: a two-round online Delphi survey was conducted to prioritize and achieve consensus on the most essential competencies for epidemic and pandemic preparedness in the MENA context. Both rounds were completed anonymously to minimize influence among experts. Experts provided iterative feedback, ensuring a refined and contextually appropriate set of competencies.
3. Third Round Consensus Meeting: a virtual consensus meeting was held to further refine and validate the competencies to be integrated into the certificate program, during which participants were identifiable.

In each round, experts reviewed and rated the list using four categories: core, essential, basic, and omitted. Core competencies were defined as the most essential or fundamental competencies required for the online course, for which the learner is expected to demonstrate exceptional knowledge and skill. Essential competencies referred to those that are important for students to master in order to be successful in the course, with learners expected to demonstrate a solid understanding and ability to apply the competency. Basic competencies were those that would be beneficial for students to possess but are not necessarily critical for success in the online course, and for which learners demonstrate a basic understanding. Omitted competencies were defined as competencies that are not critical to the course or not a priority to be incorporated and should therefore not be included in the curriculum.

Consensus was defined as ≥70% agreement among experts, a threshold widely used in Delphi studies to indicate substantial expert consensus (18). An additional 80% rule for adjacent categories was applied to capture near-consensus on borderline items. Competencies that did not reach these thresholds were re-rated in the subsequent round, with adjacent-category cases restricted to binary choice to ensure convergence.

The Delphi panel included experts in epidemic and pandemic preparedness, comprising public health professionals, government officials, emergency response managers, risk communicators, and civil society leaders. Data collection for both Delphi survey rounds, and the consensus meeting was conducted online to facilitate broad participation from experts across the MENA region.

By synthesizing findings from both the TNA survey and the Delphi process, this study offers evidence-based recommendations for the development of a competency-based certificate program that addresses the specific training needs of professionals in epidemic and pandemic preparedness in the MENA region.

### 2.3 Data analysis

Survey responses were exported into Stata SE where the data was managed and cleaned. For the Training Needs Assessment (TNA), descriptive statistics were graphed using Power BI application. Aiming to explore the statistically significant difference between score A (importance of activity to job) and score B (ability of respondent to perform the activity), as recommended by the Hennessey-Hicks manual, a parametric t-test was deployed with 95% confidence level. A total of 265 participants accessed and submitted the questionnaire. However, 170 responses did not include ratings for any of the five domain components and were therefore excluded from the analysis. The final analytical sample consisted of 95 participants who provided ratings for at least one domain. Missing data were handled using case-wise deletion. Responses without ratings for the main outcome variables could not contribute to the analysis of training priorities and were therefore excluded.

Open-ended responses from the TNA were analyzed thematically using a content analysis approach without a predefined coding framework. Two researchers independently reviewed all responses, generated short descriptive codes, and organized them into broader themes reflecting barriers, challenges, and training needs. After independent coding, the researchers met to discuss and merge overlapping codes where appropriate, reaching consensus on the final set of codes and themes. Frequencies were calculated as the number of responses assigned to each theme divided by the total number of coded responses.

### 2.4 Ethical considerations

This study was approved by the Institutional Review Board at the American University of Beirut (AUB-IRB, Protocol #SBS-2022-0256). Participation in both the TNA survey and the Delphi survey was voluntary, and informed consent was obtained prior to data collection. Data collected through the TNA survey were anonymous, and no identifying information was stored. For the Delphi process, rounds 1 and 2 were conducted confidentially, with participants blinded to one another’s identities and individual responses to maintain independent ratings. Reported data does not contain any identifiable or personal information. All data were stored on password-protected systems accessible only to the research team.

## 3 Results

### 3.1 Training needs assessment findings

#### 3.1.1 Participants characteristics

A total of 95 respondents completed the questionnaire, consisting of 46 males (45%) and 49 females (55%), with an average age of 49.6 years. Approximately 30.5% had less than 10 years of professional experience, while 23.2% had over 30 years. Nearly half of the participants specialized in public health (48%), followed by clinicians (35%), emergency managers (6.8%), researchers (2%), and medical engineers (1.2%). Respondents represented several countries across the MENA region, primarily Lebanon (27.7%), Jordan (13.3%), Qatar (10.8%), Morocco (7.2%), Egypt (6%), and Palestine (4.8%), with smaller contributions from Oman (3.6%), Mauritania (1.2%), and Bahrain (3.6%).

#### 3.1.2 Assessing training needs

**Figure 1:**
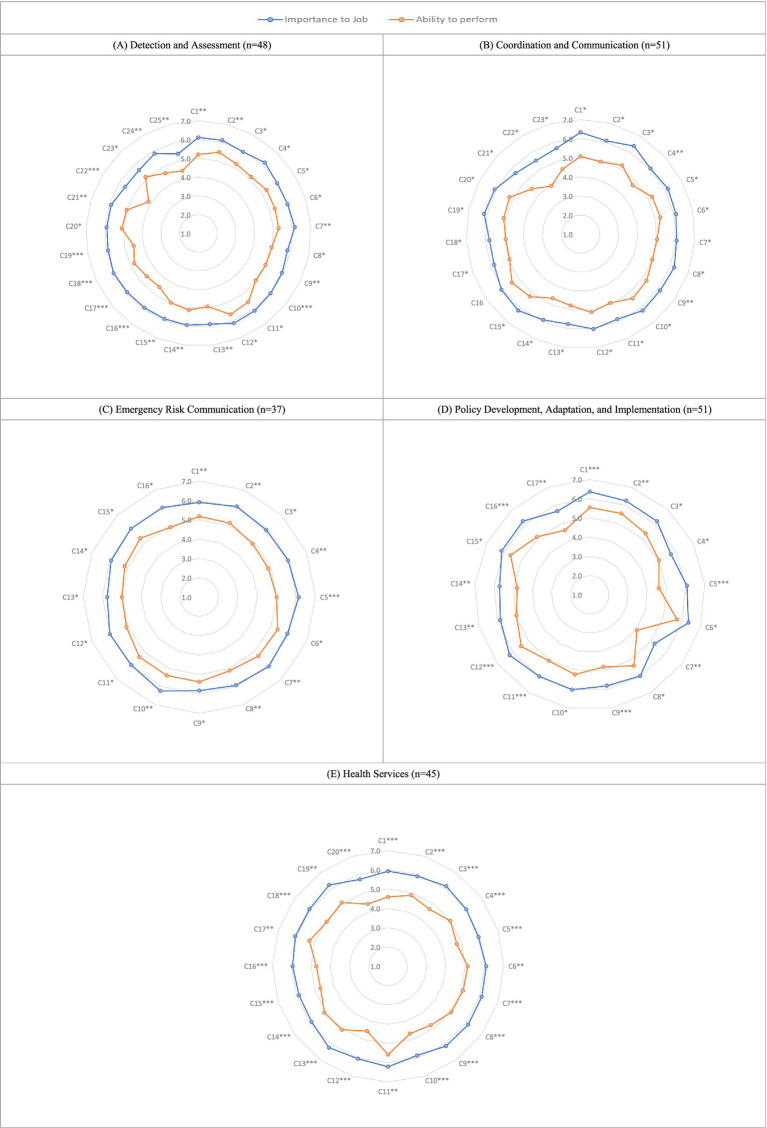
Radar plots of importance to job and ability to perform across domains: **(A)** Detection and Assessment, **(B)** Coordination and Communication, **(C)** Emergency Risk Communication, **(D)** Policy Development, and **(E)** Health Services.

Participants rated tasks related to epidemic and pandemic preparedness on a seven-point scale based on their importance to the job (Score A) and the individual’s ability to perform each task (Score B). The difference between these two mean scores was used to determine training priorities. The assessment covered five domains: detection and assessment (25 competencies), coordination and communication (23 competencies), emergency risk communication (16 competencies), health services (17 competencies), and policy development, adaptation, and implementation (20 competencies). Significant differences (*p* < 0.05) were observed across all domains, indicating widespread training needs. The most pronounced competency gaps (*p* < 0.0001) were found in the domains of health services, policy development, adaptation, and implementation, and detection and assessment, identifying them as priority areas for capacity strengthening. In contrast, comparatively smaller gaps were observed in coordination and communication and emergency risk communication (Figure 1). Detailed numerical results, including mean importance scores, mean capacity scores, and mean differences, are provided in the Supplementary material 3.

##### 3.1.2.1 Detection and assessment domain

Within this domain, 24% of competencies (six competences) demonstrated highly significant gaps (*p* < 0.0001). The most critical training needs were related to communicating findings with emergency decision-makers, establishing sensitive and real-time electronic surveillance systems, and developing sustainable border screening plans for pathogens of international concern. These findings underscore the need for targeted training to enhance emergency risk management, strengthen public health surveillance systems, and ensure compliance with international standards for disease reporting and control (Figure 1A).

##### 3.1.2.2 Policy development, adaptation and implementation domain

The results revealed substantial gaps, with 35% of competencies (six items) showing highly significant differences. Key weaknesses were identified in evidence-based policy formulation, intersectoral coordination, and collaboration with interdisciplinary specialists in infection control. Additional gaps included planning for vaccine storage, stockpiling, and regulatory approvals; preparing for both medical and non-medical countermeasures; standardizing case definitions and reporting systems; and strengthening laboratory surge capacity through the training of scientists in rapid testing procedures. The findings also underscored the necessity of enhancing the utilization of epidemiological data to inform trade and travel restrictions and to foster stronger integration between technical experts and policymakers at the national level.

##### 3.1.2.3 Health services domain

Health services showed the most significant training needs, with 80% of competencies differing highly (*p* < 0.0001). Key concerns include pre-event planning for vaccine and countermeasure stockpiles, using surveillance data to anticipate health events, and implementing effective mass vaccination and prophylaxis distribution plans. Respondents also identified critical gaps in planning, coordination, and resource management, especially in developing medical surge plans, resource-sharing strategies, and staffing processes for credentialing, compensation, and crisis support. Strengthening clinical and laboratory capacity by training scientists in rapid testing procedures and establishing specialized units for highly infectious diseases is also crucial. Additionally, planning for demobilization and recovery after emergency response operations is essential. These findings underscore urgent and systemic needs in health service delivery, workforce preparedness, and continuity planning for epidemic and pandemic response in the MENA region.

#### 3.1.3 Emerging themes on barriers, challenges, and training needs

**Figure 2:**
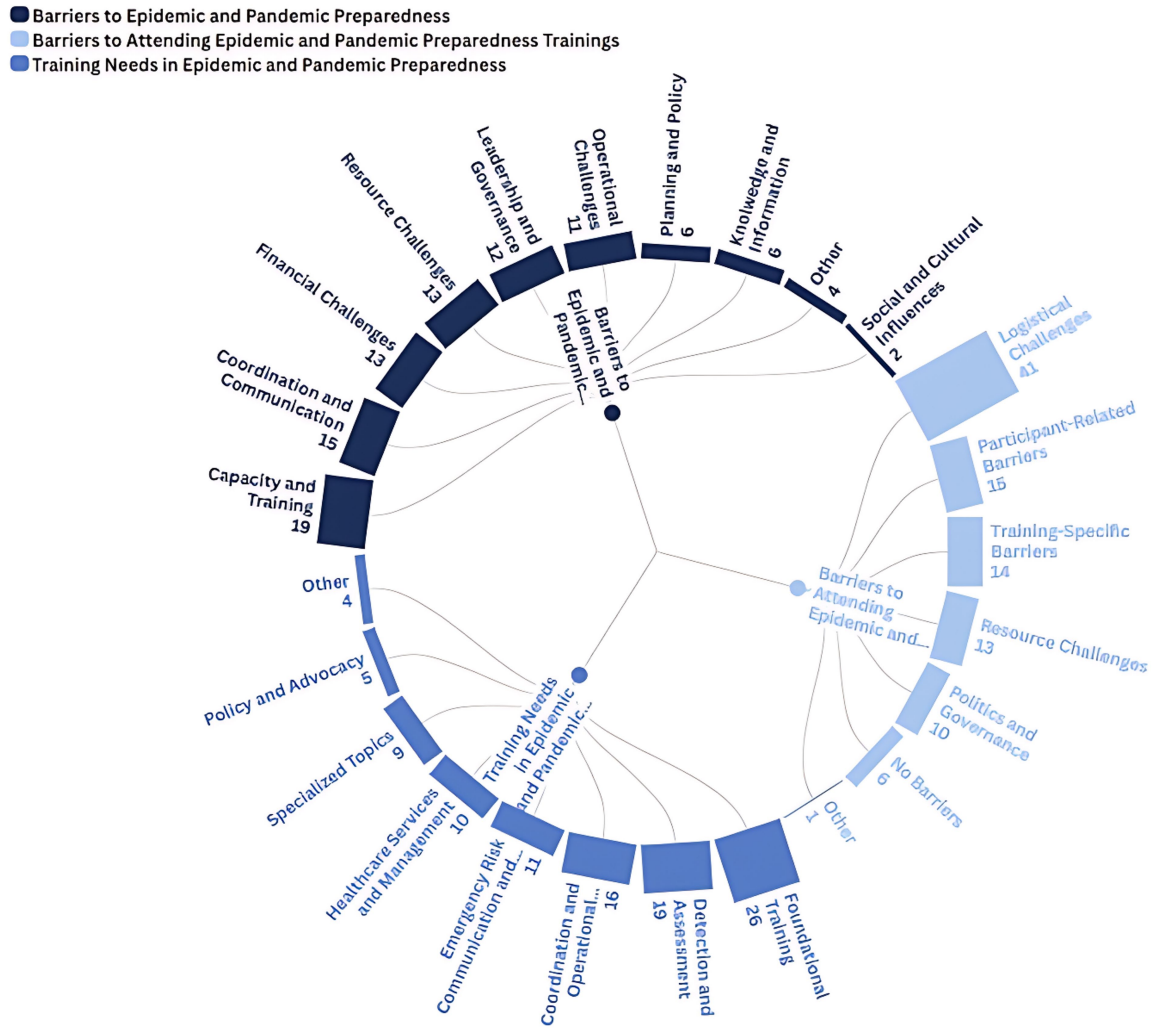
Radial bar chart showing the percentage distribution of qualitative themes from the TNA.

The qualitative analysis of open-ended responses provided valuable insights into the barriers to epidemic and pandemic preparedness, challenges in attending relevant training, and the specific capacities needed for future capacity building. These findings revealed recurring themes that align with existing literature and provide a lens into the systemic, institutional, and individual-level constraints within the MENA region (Figure 2). The results are organized around three key areas corresponding to the survey questions.

##### 3.1.3.1 Barriers to epidemic and pandemic preparedness

Capacity and training-related challenges emerged as the most significant barrier, accounting for 19% of responses. This was followed by coordination and communication challenges, which accounted for 15%. Participants frequently mentioned the absence of simulation exercises, limited technical expertise, a shortage of qualified personnel, and a lack of structured training opportunities. High staff turnover, particularly within the ministries of health, and the appointment of inexperienced or unqualified individuals to technical or leadership positions were also commonly reported. Coordination and communication barriers centered around weak inter-institutional collaboration, poor data and information sharing, and unclear roles and responsibilities. Respondents noted overlapping authorities, limited coordination among stakeholders, and weak communication both within health systems and with external actors. Resource-related and financial barriers each represented 13% of responses. Participants described shortages in supplies, materials, and personnel, alongside high turnover rates. Financial limitations, including insufficient funding, budget constraints, and broader economic crises in several countries, were also viewed as major obstacles to preparedness. Leadership and governance barriers accounted for 12% of responses and encompassed themes of political interference, weak governance structures, leadership gaps, and corruption, reflecting deeper systemic challenges. Operational barriers (11%) were linked to technological and logistical difficulties, weak surveillance infrastructure, and inadequate systems for emergency operations. Less frequently reported barriers included deficits in planning and policy (6%), limited knowledge and information sharing (6%), and social and cultural influences (2%). These factors were noted to affect public engagement and trust in preparedness initiatives.

##### 3.1.3.2 Barriers to attending epidemic and pandemic preparedness trainings

When asked about obstacles to participating in training programs, most respondents cited logistical challenges, particularly heavy workloads, time constraints, and transportation difficulties. These factors limited their ability to attend or prioritize additional training. Participant-related barriers accounted for 15% of responses, including a lack of awareness about available training opportunities, low perceived need, and limited motivation for further education. Training-specific barriers, which accounted for 14%, often compounded these challenges. Respondents described insufficient course availability, lack of incentives to participate, and concerns about the quality and contextual relevance of training content.

Resource-related barriers, which accounted for 13%, included both financial and human resource shortages. Participants noted that economic hardship and training costs limited attendance, while institutions often lacked the necessary expertise to deliver specialized instruction. Politics and governance-related barriers, which accounted for 10%, were associated with policy gaps, competing interests, power imbalances, and political interference. These factors all constrained access to preparedness training. Interestingly, 6% of respondents reported no barriers, suggesting that in certain contexts, access to training and capacity-building opportunities remains relatively unimpeded.

##### 3.1.3.3 Training needs in epidemic and pandemic preparedness

Participants expressed a wide range of training interests encompassing detection and assessment, healthcare services and management, policy and advocacy, coordination and operations, and emergency risk communication. Foundational training received the highest response percentage (26%), followed by detection and assessment (19%) and coordination and operations (16%). Within detection and assessment, respondents highlighted the necessity of training in risk assessment, infectious disease surveillance, and risk management. Interest in coordination and operational training focused on logistics, response management, planning, and intersectoral collaboration.

Training related to healthcare services and management (10%) and emergency risk communication and community engagement were also frequently mentioned, particularly in the context of infection prevention and control, critical care preparedness, and vaccination planning. Smaller proportions of participants emphasized the importance of training in policy development and advocacy (5%) and specialized topics (5%) such as One Health approaches and statistical or analytical tools. Collectively, these findings demonstrate a broad spectrum of training needs across multiple domains and underscore the region’s interest in both foundational and advanced preparedness competencies.

### 3.2 Delphi survey findings

The Delphi process, comprising three rounds, aimed to reach expert consensus on the core competencies for epidemic and pandemic preparedness in the MENA region. Data analysis was conducted using SPSS version 26 software. Descriptive statistics, including counts and percentages, were employed to assess the level of consensus for each competency.

In Round 1, competencies that received ≥70% agreement in any of the three categories (Core, Essential, or Basic) were considered to have reached consensus Competencies receiving exactly *70%* agreement as omitted were to be excluded due to insufficient support. Additionally, competencies that achieved an aggregated agreement 80% or more across two adjacent categories (either *Core and Essential* or *Essential and Basic*) were retained for Round 2 for further evaluation within those narrowed options and applying the same consensus guidelines as round 1. Competencies that did not reach consensus in Round 2 were discussed and re-evaluated in a final consensus meeting (Table 1).

**Table 1:** Delphi rounds and consensus progression.

Round	Reached ≥70% consensus	Combined 80% agreement		Remaining re-rating/removal
		Core and essential	Essential and basic	
Round 1	20	44	15	20
Round 2^¥^	58	28	9	4
Round 3^¥^	99	–	–	–

^¥^Rounds 2 and 3 include competencies carried forward from earlier stages.

#### 3.2.1 Participant characteristics

A total of 10 experts, representing diverse professional and geographic backgrounds, participated in the Delphi process. These experts were drawn from Bahrain, Egypt, Syria, Lebanon, the United Arab Emirates, Jordan, and the United Kingdom. They included faculty members, professors, advisory board consultants, medical doctors, public health program directors, and infectious disease specialists. Participation rates varied across the rounds. In Round 1, 10 experts responded, while in Round 2, the number decreased to 8. The consensus meeting, round 3, saw a further reduction in participation to 5. Notably, five participants completed all three rounds, ensuring continuity and informed decision-making throughout the iterative process.

#### 3.2.2 Round I results

The first-round questionnaire was distributed via email to 25 invited experts, with 10 responding (40% participation rate). Experts reviewed 99 competencies spanning five main domains: detection and assessment, coordination and communication, emergency risk communication, health services, and policy development, adaptation, and implementation.

In this round, 20 competencies achieved the predefined consensus threshold (≥70%) for classification as *core*, *essential*, or *basic*. An additional 59 competencies achieved partial agreement across adjacent categories (*core/essential* or *essential/basic*), while 20 competencies did not reach consensus and were flagged for re-rating or removal in subsequent rounds.

#### 3.2.3 Round II results

In Round 2, eight of the ten Round 1 experts participated. During this stage, consensus was reached on an additional 38 competencies, bringing the cumulative total to 58 competencies classified with clear agreement. Among these, 28 competencies were classified as Core or Essential, 9 as Essential or Basic, and 4 remained inconclusive, requiring further discussion in the final consensus round.

#### 3.2.4 Round III results

The third and final round involved a consensus meeting in which participating experts re-rated the remaining competencies: 28 falling between Core and Essential, 9 falling between Essential and Basic, and the 4 additional competencies that were still inconclusive. Through structured discussion, the panel finalized agreement on the remaining items, resulting in a total of 99 competencies across all domains. Agreement was ultimately reached that the four remaining inconclusive competencies were Essential, covering the following key areas: (1) Detection and Assessment: consensus emphasized the ability to interpret information from existing surveillance systems to characterize affected populations, monitor disease trends, and evaluate control measures; (2) Policy Development, Adaptation, and Implementation, experts agreed on the importance of regularly reviewing, testing, and updating standard operating procedures prior to response activation, and ensuring the availability of a multi-sectoral task force for coordination during emergencies for effective coordination and integration across relevant sectors during response operations; (3) Health Services, agreement was reached on the need for collaboration with clinicians to develop medical surge plans tailored to diverse public health threats; (4) Coordination and Communication, two key competencies were highlighted: conducting regular multidisciplinary simulation exercises to enhance communication among staff and partners, and proactively identifying and addressing flawed assumptions in response plans before activation.

**Figure 3:**
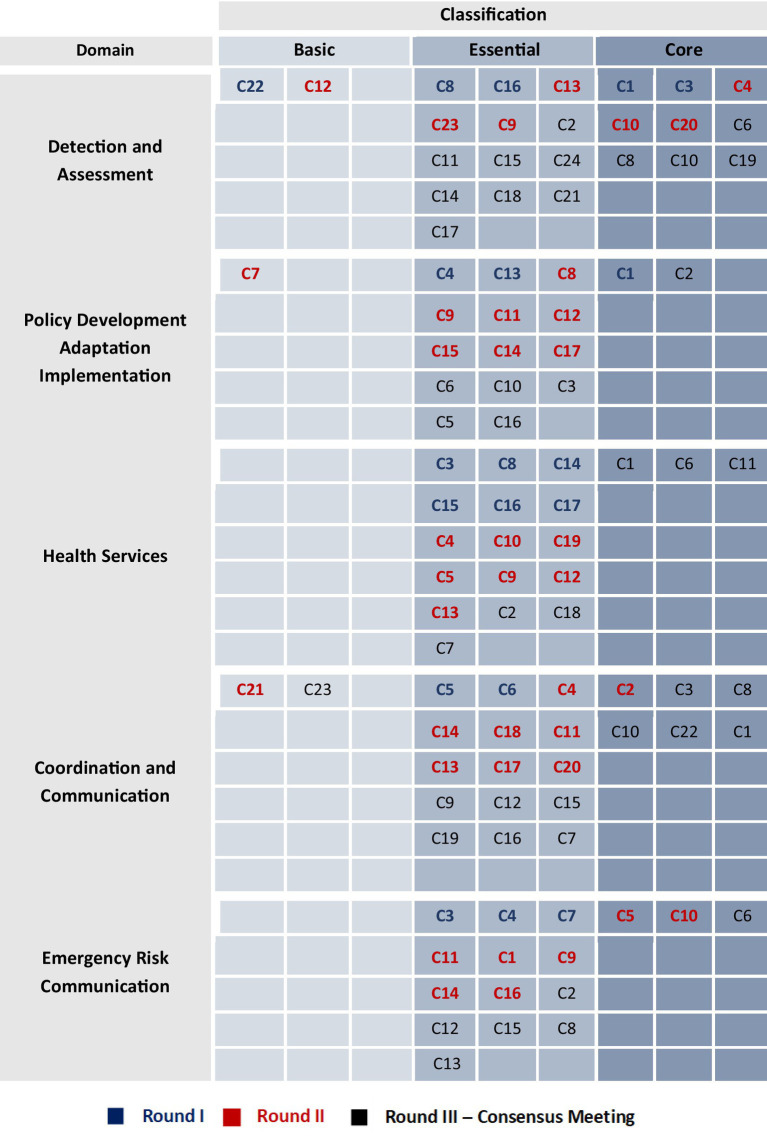
Final classification of competencies across the five preparedness domains. *No competencies were omitted from the analysis. Of the total, only 5 were classified as basic, while the majority were classified as essential (71 competencies). The remaining competencies were classified as core (23 competencies). Most of the core competencies were concentrated within the Detection and Assessment and Coordination and Communication Domains.

All the 99 competencies were finalized in Round 3 and were considered crucial components of preparedness capacity as their importance level is shown in Figure 3.

## 4 Discussion

This study identified critical gaps in epidemic and pandemic preparedness in the MENA region, with the TNA results demonstrating that most competencies across all five domains revealed a gap, highlighting a growing need for capacity building within the workforce. The most notable gaps identified were in the domains of Detection and Assessment and Health Services. The findings underscore the urgent need for context-specific, competency-based training to strengthen preparedness in the MENA region. While the broad structural challenges that exist in many low- and middle-income countries (LMICs) contribute to these weaknesses, the MENA region, marked by protracted conflicts, economic fragility, and displacement crises likely intensify the gaps observed in our findings (19). Comparable regional assessments have also emphasized the importance of strengthening surveillance systems, health system capacity, workforce development, and strategic planning as core priorities for pandemic preparedness (20).

The most significant need was observed in the health services domain, placing it as the most vulnerable point in epidemic and pandemic preparedness. Participants reported gaps in medical surge capacity planning, vaccine and prophylactic medication distribution, and retention of trained healthcare personnel. Identified needs align with regional evidence suggesting shortages in ICU beds, medical equipment, and training workforce (21). Given the resource limitations and political instability, COVID-19 exacerbated this weakness within and across countries in the region (22). Similar challenges were observed in other regions during the COVID-19 pandemic, where health systems had to rapidly expand hospital and ICU capacity while mobilizing additional health professionals to respond to increased demand for care (23). Findings also revealed a lack of access to training, lack of sufficient and experienced staff, as well as a deficit in basic foundational training in preparedness. Most low- and middle-income countries in the region lack comprehensive training pathways or sufficient staffing capacity (24). Findings also showed a need to strengthen vaccine distribution competencies, which aligns with regional challenges with uneven resources, and logistical constraints which have hindered timely rollout, even when vaccines were available (25, 26). Crucially, the high importance but low ability scores for vaccine distribution suggest that the bottleneck in the MENA region is not merely a matter of supply, but of systematic delivery. This implies that infrastructure investment alone is insufficient without a parallel focus on the logistical and surge-planning competencies of the workforce (5, 14, 27).

Beyond health services, the findings also revealed major gaps in surveillance, data systems, and early detection capacities. Skills like communicating risk assessment findings, establishing sustainable indicators and event-based surveillance, and utilizing real-time data platforms, demonstrated the largest gaps between importance and ability to perform amongst respondents. These limitations are rooted in infrastructural and resource constraints across the region. Similar challenges in surveillance and early detection capacities have also been highlighted globally. Analyses of international preparedness frameworks suggest that although countries are expected to develop systems for early detection and reporting, many still face limitations due to gaps in infrastructure, workforce capacity, and data systems (28). The literature similarly highlights the region’s longstanding struggle with fragmented evidence systems, and underinvestment in infrastructure that can support surveillance, data sharing, and data governance (29). Also observed in several MENA countries, cross-border surveillance systems face persistent issues due to lack of expertise in the field (12). These documented structural barriers likely contribute to the competency gaps identified in the TNA emphasizing the need for adequate platforms for epidemic data, coupled with training staff to prepare for and effectively respond to crisis, is essential for strengthening national and regional preparedness (30, 31).

Weaknesses within the detection and assessment domain do not exist in isolation but are rather compounded by policy limitations that can restrict multi-sectoral collaboration, planning, and timely response. Findings highlighted challenges with coordination across sectors, which would affect translating preparedness plans into operational practice and in integrating countermeasures into national preparedness frameworks. These mirror broader structural challenges documented in the MENA where policy responses to pandemics have often been fragmented due to weak regional governance and coordination (32). Similar observations have been reported in other settings, where effective pandemic response depended not only on technical capacity, but also on strong cross-sector collaboration and adaptive policy implementation across government and non-government actors (33). Qualitative findings from the study showed that power, politics, and governance were perceived as major factors when it came to barriers to epidemic and pandemic preparedness. Evidence consistently shows that policy barriers driven by political divides, conflict, and fragmented systems, limit the effectiveness of regional coordination efforts and data sharing (34, 35). The qualitative finding that power and politics are major barriers provides a causal explanation for the low coordination scores in the TNA. It suggests that technical experts feel unable to perform coordination tasks not necessarily due to a lack of theoretical knowledge, but because the ‘securitized’ nature of regional health governance, where response is often led by political actors, effectively excludes public health professionals from the strategic decision-making process (25, 34).

These issues extended into coordination and emergency risk communication, which also showed notable gaps. Coordination during health crises in the MENA region remains limited, as multisectoral committees often lack clear mandates, structured decision-making, and sustained collaboration (36). Engagement between sectors was often limited to information sharing rather than coordinated planning or implementation (19). Emergency risk communication also remains an underdeveloped skill, with gaps identified in areas related to risk communication, and community level engagement strategies. Communication plans in many countries were neither clearly defined within government structures nor designed to reach diverse populations in a timely, culturally appropriate manner (19). Effective epidemic communication also requires clear, consistent messaging and engagement with community stakeholders, yet studies have shown that misinformation, inconsistent messaging across institutions, and limited collaboration between health actors frequently undermine these efforts (37). This points to the need for competencies that integrate strategic communication planning, and to invest in capacity building for communication specialists (14).

The Delphi process further strengthened the TNA findings by validating and prioritizing competencies that regional experts considered most critical for preparedness training. Consensus was reached gradually, with only 20 competencies reaching consensus in Round 1, reflecting the diverse contexts and operational realities across the MENA region. This slow convergence underscores the difficulty of applying universal standards across a region split between high-resource Gulf states and conflict-affected nations. Furthermore, the experts’ classification of most competencies as “Core” or “Essential” rather than “Basic” indicates a high regional demand for advanced technical and operational proficiency. Ultimately, the Delphi findings reinforce that strengthening the regional workforce is essential, and many competencies need to be enhanced to address the existing gaps.

Together, the TNA and Delphi findings provide a clear direction for strengthening epidemic and pandemic preparedness across the MENA region. They highlight not only where the most critical gaps lie, but also the level of competency required for an effective and sustainable regional preparedness workforce. This informed the development of a regional, context-specific certificate that reflects the structural differences and disparities across the MENA region. Considering the regional landscape and challenges, time constraints, and instability, an online asynchronous format allows learners from diverse settings to develop their skills and competencies. Grounding the curriculum in the competencies validated through the study ensures that the program directly addresses the needs of the region and ensures a more resilient workforce.

### 4.1 Strengths and limitations

A significant strength of this study is its mixed-methods design, combining TNA with a Delphi process to inform a contextualized certificate in epidemic and pandemic preparedness. By engaging public health professionals with experience in the MENA region in several Delphi rounds, the study confirmed and refined priorities in capacity building needs. This aligns with best practices in public health workforce development, where structured, iterative expert feedback through a Delphi survey is used to refine competency frameworks (38). The results provide a strong foundation for designing a certificate program that is both evidence-based and relevant to the specific challenges faced in the MENA region.

While the study provides valuable insights, several limitations should be considered. The TNA relied on self-reported data, which may introduce response bias. Additionally, the response rate in the Delphi rounds declined, with the final consensus meeting consisting of six experts, potentially limiting the number of perspectives. However, the remaining experts represented diverse geographic and institutional backgrounds, helping mitigate this limitation. Future studies should aim to replicate the Delphi process with larger samples or include follow-up validation workshops to confirm identified competencies.

## 5 Conclusion

This study represents a systematic effort to develop and validate context-specific competencies for epidemic and pandemic preparedness in the MENA region. By integrating empirical training needs with expert consensus, the study bridges the gap between global frameworks and regional realities. Despite the training needs identified in all domains, the most significant gaps were observed in the detection and assessment, health services, policy implementation, and communication and coordination domains. The use of both the TNA and Delphi provides a strong base for establishing competencies that are relevant to regional challenges and grounded in expert feedback. The resulting competency framework forms the foundation for a MENA-specific preparedness certificate program, designed to strengthen workforce capabilities, enhance health system resilience, and promote sustainable regional collaboration. Implementation of this program across public health institutions could significantly improve the region’s readiness for future outbreaks and emergencies.

## Data Availability

The original contributions presented in the study are included in the article/Supplementary material, further inquiries can be directed to the corresponding author.

## Supplementary material

SUPPLEMENTARY MATERIAL 3 Training Needs Assessment (TNA) scores across competencies, including importance to job, ability to perform, and calculated differences by domain.
