# Supplementary material for: Developing context-specific competencies for epidemic and pandemic preparedness in the MENA region: a training needs assessment and Delphi approach
Source: Front Public Health. 2026 Apr 20;14:1778190. doi: 10.3389/fpubh.2026.1778190 (PMC13136252; doi:10.3389/fpubh.2026.1778190)
Supplement: SUPPLEMENTARY MATERIAL 3 — Training Needs Assessment (TNA) scores across competencies, including importance to job, ability to perform, and calculated differences by domain. [file Data_Sheet_1.PDF]

Supplementary Material: Competencies Across Five Domains

Detection and Assessment

The main functions of detection and assessment capabilities are to (1) enable preparedness systems to recognise and characterise a threat, (2) monitor its impact on the population, and (3) evaluate the efficacy of interventions to control the threat. Assessment depends on having laboratory and surveillance capacities in place before an event; this includes appropriate legal arrangements. Information flowing from assessment activities must be communicated to all segments of the public health preparedness system, as well as with the public, to support policy development and implementation, prevention and treatment efforts.

**C1**	Using event-based and indicator-based surveillance system to detect health threats.
**C2**	Know when case reports or clusters require further investigation, and how to initiate such investigation.
**C3**	Evaluate the implications of national or international public health alerts.
**C4**	Identifying as rapidly as possible the (possibly novel) agents responsible for a disease outbreak and their epidemiological characteristics
**C5**	Update estimates of an agent's epidemiologic characteristic as new information becomes available
**C6**	Characterize the current and potential human health consequences of population exposure to the threat.
**C7**	Perform a risk assessment.
**C8**	Apply the results of international risk assessments to your country
**C9**	Communicate the results and implications of risk assessments to policymakers with different backgrounds
**C10**	Communicate the results and implications of risk assessments to those responsible for emergency risk communication.
**C11**	Develop case definitions to validate and analyze case reports.
**C12**	Conduct outbreak investigations to identify pathogens, characterize affected population groups, and sources of exposure
**C13**	Conduct case-control studies and other epidemiologic studies to test hypotheses regarding sources of exposure.
**C14**	Collaborate with local health officials, healthcare providers, and others to conduct outbreak investigations and epidemiologic studies
**C15**	Collaborate with international organizations to conduct coordinated multinational epidemiologic studies.
**C16**	Establish and maintain indicator and event-based surveillance system(s) to detect public health threats.
**C17**	Establish and maintain electronic real-time reporting systems
**C18**	Interpret information from existing surveillance in order to characterize affected population groups, and to monitor disease trends and the impact of control strategies.
**C19**	Develop and implement plans for border screening for known pathogens of international concern.
**C20**	Conduct timely and accurate disease reporting in accordance with WHO requirements
**C21**	Collaborate with local public health officials and the healthcare delivery system, initiate active surveillance to identify additional cases during an epidemiologic investigation.
**C22**	Manage a national laboratory system and effective modern point-of-care and laboratory-based diagnostics
**C23**	Participate in multinational epidemiologic studies
**C24**	Have the biological, clinical, and epidemiological knowledge needed to characterize (potentially novel) pathogens and other agents responsible for an outbreak disease.
**C25**	Integrate and interpret information from a variety of local, national, and international sources regarding contaminants in air, soil, and water.

Policy Development, Adaptation, and Implementation

The main functions of policy development, adaptation and implementation capabilities are to (1) adapt existing authorities and policies to the new and emerging circumstances of a cross-border threat to health, and (2) enforce existing and new laws and regulations needed to implement these policies and regulations. This section mainly deals with the development and implementation of substantive policies, regulations, and official guidance regarding infection control and disease treatment in clinical settings to population-based disease control activities at the national level.

**C1**	Work with epidemiologists, microbiologists, environmental sciences and others to continuously evaluate evidence on patient treatment and infection control.
**C2**	Regularly assess and, as needed, clarify existing policies and/or recommend/advocate measures and communicate them to health officials at the ministry level, border control officials, and others.
**C3**	Share relevant information with healthcare, infection control, and patient transport experts, and solicit their feedback

C4	Seek and receive advice from public health professionals in making border control decisions and reflect to the public how and why these decisions have been made
C5	Be able to use data products from epidemiologists in providing advice in the development of trade and travel restrictions as tools of population-based disease control.
C6	Communicate the necessity of policies calling for personal protective measures to mitigate personal risks for the public health professionals.
C7	Aid the transfer of medical and related professionals across borders and facilities through standardized job descriptions of personnel in clinical settings.
C8	Provide healthcare workers with clinical guidelines for emerging infections from abroad, especially those that may be carried by travelers and the severely contagious.
C9	Before the response operation, ensure regular assessments of legal frameworks and propose/advocate measures to address gaps.
C10	Before the response operation, assess if the implementation of strategies, plans, and action plans requires any changes in these plans and strategies.
C11	Before the response operation, identify which triggers will require key decisions during outbreak response (keeping in mind that triggers may need to be modified to fit specific situations).
C12	Review the evidence on current or impending outbreaks; propose and advocate adaptations to policies as needed.
C13	Communicate policy/guidelines, weigh benefits and costs, understand concerns about implementation, and adapt policies related to border control.
C14	Continuously evaluate evidence on threats; communicate if border control policies need to be adapted.
C15	Share information with response managers and health officials at the ministry level to support decisions about appropriate countermeasures.
C16	Before response activities are taken, regularly review, test, and update the standard operating procedures and ensure that a multi-unit task force is available for the coordination and integration of relevant sectors during response operations.
C17	Before the response operation, ensure the adequacy of plans for financing and credentialing of staff during emergency situations.

Health Services

The main functions of health services include (1) the provision of vaccines and other countermeasures to healthcare workers and the general public, and (2) the physical and mental health treatment for those affected by mass-casualty or long-running incidents. The medical countermeasures, supplies and equipment needed to provide these services are considered capacities, but procuring, stockpiling, and distributing them on an emergency basis during a crisis are critical capabilities, and thus included in this section. The competencies in this section mainly deal with what must be acco

C1	Before an event, plan for the storage and stockpiling of vaccines and prepare for medical and non-medical countermeasures.
C2	Draw upon the work of surveillance networks to identify potential events that may indicate the need for the implementation of preventative services plans.
C3	Ensure that plans are in place for mass vaccinations and mass prophylactic medication distribution.
C4	Coordinate vaccination plans and criteria for vaccination target groups to ensure consistency of practices.
C5	Facilitate the approval of vaccines through streamlined processes where available.
C6	Address antimicrobial stewardship activities.
C7	Prior to an event, work in tandem with clinicians to develop medical surge plans for various threats.
C8	Ensure that plans across the continuum of care have been communicated to the clinical staff to effectively manage surge needs
C9	Plan for combining resources at national and local levels (e.g. cross-border sharing of clinicians if a hospital reaches capacity).
C10	Establish processes for staffing related surge issues including credentialing, paying staff, channels of authority, extended crisis interventions, and livelihood protection at home.
C11	Establish reliable systems for disseminating case definitions to standardize both the diagnosis and the reporting of case numbers (e.g. confirmed, suspected, probable, or possible cases).
C12	Assess laboratory capacity on an ongoing basis and train public health scientists in rapid testing procedures to ensure adequate surge capacity
C13	Create a hospital-based unit for critical, contagious patients at select facilities known to medical evacuation teams.
C14	Work with health personnel to identify the best medical countermeasures based on risk and threat; relay the results of these conversations
C15	Ensure flexible policies and procurement strategies including how to allocate resources in the event of a shortage.
C16	Ensure there are adequate levels of human resources (e.g. experts) and laboratory capacity available.
C17	Use standardized approaches to engage with all personnel who may serve in field operations on the use of PPE.
C18	Before a response operation, relay to healthcare workers the importance of their role in public health emergencies and support their personal preparedness and that of their families.
C19	Establish ways to procure PPE for medical professionals and emergency responders.
C20	Plan for the demobilization and recovery of the healthcare workforce after a response operation.

Coordination and Communication

This section covers important aspects of communication that relate to the coordination and management of the public health emergency preparedness system during a cross-border threat. The competencies and capabilities described are not ends in themselves, but rather describe how these ends can be achieved and what must be accomplished during a crisis. The focus here is on key aspects of coordination and communication.

**C1**	Continuously create and update an incident management plan that adapts existing policies to the situation at hand
**C2**	Continuously inform public health emergency response managers about the threat so that the incident management plan can be updated.
**C3**	During the response operation, anticipate resource needs and communicate them to relevant decision makers
**C4**	Before the response operation, practice and test the ability to make decisions under uncertainty.
**C5**	Participate in the implementation of plans which ensure the continuity of operations.
**C6**	Communicate with political decision makers to mobilize needed resources, communicate current knowledge and uncertainties, and solicit guidance.
**C7**	Before the response operation, identify key assumptions behind plans, identify untenable assumptions, and advocate changes as needed.
**C8**	Develop protocols and test/exercise processes for health emergency operations and their activation.
**C9**	Before the response operation, establish rapid communication channels within national disease surveillance and healthcare professionals.
**C10**	Before the response operation, establish trust with healthcare providers through feedback loops and two-way communication.
**C11**	For incident communication, draw on clinical personnel trained in risk communication or people involved in the incident, such as doctors or other clinicians.
**C12**	Provide training; include healthcare providers in drills and exercises to test communication lines and avoid communication problems.
**C13**	Before the response operation, ensure that key partners are familiar with applicable laws, key roles, resources, information needs, and planning assumptions.
**C14**	Before the response operation, ensure adequate preparations for implementing health screening at borders; also ensure that response measures to a public health emergency can be taken right at the point of entry
**C15**	Advocate the development of plans for joint task forces or other entities which can share information across disciplines.
**C16**	Advocate regular multi-discipline exercises to improve communication with staff and partners.
**C17**	Before the response operation, review mutual aid agreements (where relevant), identify gaps, and propose/advocate solutions to address gaps.
**C18**	Before the response, train staff members in confidentiality policies, chains of evidence, and security issues relating to the exchange of information between partner organizations.
**C19**	Identify key partners and develop a common understanding of roles, resources, planning assumptions, risks/vulnerabilities, and information that should be shared during response operations
**C20**	Develop strategies to communicate with professionals who have different skills and knowledge levels; develop strategies to communicate with partner organizations
**C21**	Advocate regular multi-country exercises to improve the ability to communicate with partners.
**C22**	Assess the quality of the microbiology networks.
**C23**	Assess the adequacy of mutual aid mechanisms and multi-disciplinary taskforces.

Emergency Risk Communication

Emergency risk communication is the real-time exchange of information, advice and opinions between experts and/or officials, and people who face a threat to their survival, health, economic or social well-being. The focus of this section is on communicating with the public during a crisis, ensuring that everyone, especially members of vulnerable and hard-to-reach populations, receive the information that they need to protect themselves.

**C1**	Address cultural and societal barriers in the cognitive processing and compliance with recommended behaviors.
**C2**	Use most appropriate content and trusted channels of communication across population groups.
**C3**	Identify strategies to overcome linguistic barriers, e.g. request local assistance
**C4**	Identify data gathering mechanisms to understand and monitor the informational needs of the population
**C5**	Prevent and counter misinformation.
**C6**	Proactively address the needs of the news media and the general public.
**C7**	Integrate the results of the risk-assessment process in the messages.
**C8**	Manage and assess situational information received by the organization.
**C9**	Anticipate questions from the public and develop appropriate answers.
**C10**	Understand and implement the principles of risk communication
**C11**	Identify strategies to facilitate the release of information (i.e. review outgoing messages in a timely manner).

**C12**	Understand laws and regulations related to emergency risk communication.
**C13**	Provide information to the public on the roles and responsibilities of the various organizations involved in the response operation; try to understand the public's perception of the emergency
**C14**	Identify strategies to engage with government leaders in order to integrate government priorities and community interests; address concerns that surface during the emergency response.
**C15**	Identify communication mechanisms that are trusted by the public, partners, and community influencers.
**C16**	Empower the public to participate in open discussions; involve the public in decisions relevant to public health threats.
