# Supplementary material for: Developing context-specific competencies for epidemic and pandemic preparedness in the MENA region: a training needs assessment and Delphi approach
Source: Front Public Health. 2026 Apr 20;14:1778190. doi: 10.3389/fpubh.2026.1778190 (PMC13136252; doi:10.3389/fpubh.2026.1778190)
Supplement: Supplementary file 2 [file Data_Sheet_2.PDF]

Annex 4

Epidemic and Pandemic Preparedness - Training Needs Assessment

The Epidemic and Pandemic Preparedness Program (EPaPP) aims to generate and promote contextualized evidence on improving equitable and responsive epidemic and pandemic preparedness in the Middle East and North Africa (MENA) region by enhancing workforce capacity, collaborations, information systems, and governance mechanisms.

The aim of this survey is to assess the training needs, barriers, and priorities related to epidemic and pandemic preparedness in the MENA region among relevant stakeholders.

There are 27 questions in this survey.

Demographics

Country of residence *

Please write your answer here:

Gender *

Choose one of the following answers

Please choose **only one** of the following:

- Male
- Female
- Prefer not to disclose
- Other

Age *

Only numbers may be entered in this field.

Please write your answer here:

Select the workforce category related to epidemic/pandemic preparedness that best describes your field of work. *

□ Check all that apply

Please choose **all** that apply:

- ☐ Health officials at the ministry level
- ☐ National and subnational public health agency leaders
- ☐ Liaison with local public health institutions
- ☐ National focal point for preparedness and response
- ☐ Public health epidemiologists
- ☐ Public health microbiologists
- ☐ Physicians and communicable disease control specialists
- ☐ Healthcare infection control experts
- ☐ Environmental scientists
- ☐ Public health veterinarians
- ☐ Public-sector healthcare delivery system managers
- ☐ Liaison with non-governmental hospitals and health professionals
- ☐ Officials responsible for procurement and management of medical products and technology
- ☐ Regulatory agency leadership
- ☐ Public health legal advisors
- ☐ Public health emergency response managers
- ☐ Public health emergency preparedness planners
- ☐ Risk communicators
- ☐ National focal point for public health communication
- ☐ Civil protection agency leadership
- ☐ Civil society leadership
- ☐ Other: ____________

Do you think there should be other workforce categories specific to your setting?

*

Please choose **only one** of the following:

- ◯ Yes
- ◯ No

Please list the suggested workforce categories. Please feel free to add an additional line by clicking on the 'Add Line' button if needed.

Only answer this question if the following conditions are met:
Answer was 'Yes' at question ' [G00Q34]' ($(document).on('ready pjax:scriptcomplete',function(){ // Define some text strings var originalModalText = "One or more Required Questions have not been answered. You cannot proceed until these have been completed."; var newModalText = "Please notice you haven't answered this question. Still, you can continue without answering."; // Modify the modal text var modalBody = $('.modal-body p').filter(function() { return $.trim($(this).text()) == originalModalText; }); if(modalBody.length > 0) { modalBody.text(newModalText); } }); Do you think there should be other workforce categories specific to your setting?)

Type of Institution you are currently employed in *

 Choose one of the following answers
Please choose **only one** of the following:

- ◯ Educational
- ◯ Health Sector
- ◯ Governmental
- ◯ Humanitarian
- ◯ Other ____________

Years of experience in epidemic and pandemic preparedness *

 Only numbers may be entered in this field.
Please write your answer here:

Training Needs Assessment

The following training needs assessment will be done based on the expertise categories below. Please check one (or more) category that is most suitable for your expertise. *

 Check all that apply
Please choose **all** that apply:

☐ **Detection and Assessment:** *Incident recognition, risk characterization, epidemiological investigation, surveillance and epidemiological monitoring, laboratory analysis, environmental monitoring.*

☐ **Policy Development, Adaptation, and Implementation:** *Infection control and treatment guidance, population-based disease control, communicating between national and subnational authorities and enforcing laws and regulations.*

☐ **Health Services:** *Preventive services, medical surge, management of medical countermeasures, supplies and equipment, medical services for healthcare workers and emergency responders.*

☐ **Coordination and communication (within the public health emergency preparedness system):** *Crisis management, communication with healthcare providers, communication with emergency management, public safety, and other sectors, communication with other public health agencies at the global, national, and subnational levels.*

☐ **Emergency risk communication (with the public):** *Address communication inequalities, use dynamic listening and manage rumors, communicate risk in an accurate, transparent and timely manner, foster and maintain trust.*

Detection and assessment. Workforce groups include but are not limited to: environmental scientists, national public health agency leaders, public health microbiologists, national focal point for preparedness, public health epidemiologists, national public health agency leaders

Please rate the following activities according to the following criteria.

- (A) How important is this activity to the successful performance of your job? not at all important = 1; very important = 7

- (B) How well do you consider that are you able to perform the activity? not at all = 1; very proficient = 7

*

Only answer this question if the following conditions are met:
Answer was at question ' [G04Q23]' ($(document).on('ready pjax:scriptcomplete',function(){ // Define some text strings var originalModalText = "One or more Required Questions have not been answered. You cannot proceed until these have been completed."; var newModalText = "Please notice you haven't answered this question. Still, you can continue without answering."; // Modify the modal text var modalBody = $('.modal-body p').filter(function() { return $.trim($(this).text()) == originalModalText; }); if(modalBody.length > 0) { modalBody.text(newModalText); } }); The following training needs assessment will be done based on the expertise categories below. Please check one (or more) category that is most suitable for your expertise.)

	**Importance of a job (A)**	**Ability to perform (B)**
**Using event-based and indicator-based surveillance system to detect health threats.**		
**Know when case reports or clusters require further investigation, and how to initiate such investigation.**		
**Evaluate the implications of national or international public health alerts.**		

	Importance of a job (A)	Ability to perform (B)
**Identifying as rapidly as possible the (possibly novel) agents responsible for a disease outbreak and their epidemiological characteristics.**		
**Update estimates of an agent's epidemiologic characteristic as new information becomes available.**		
**Characterize the current and potential human health consequences of population exposure to the threat.**		
**Perform a risk assessment.**		
**Apply the results of international risk assessments to your country.**		
**Communicate the results and implications of risk assessments to policymakers with different backgrounds.**		
**Communicate the results and implications of risk assessments to those responsible for emergency risk communication.**		
**Develop case definitions to validate and analyze case reports.**		
**Conduct outbreak investigations to identify pathogens, characterize affected population groups, and sources of exposure.**		
**Conduct case-control studies and other epidemiologic studies to test hypotheses regarding sources of exposure.**		

	Importance of a job (A)	Ability to perform (B)
**Collaborate with local health officials, healthcare providers, and others to conduct outbreak investigations and epidemiologic studies.**		
**Collaborate with international organizations to conduct coordinated multinational epidemiologic studies.**		
**Establish and maintain indicator and event-based surveillance system(s) to detect public health threats.**		
**Establish and maintain electronic real-time reporting systems.**		
**Interpret information from existing surveillance in order to characterize affected population groups, and to monitor disease trends and the impact of control strategies.**		
**Develop and implement plans for border screening for known pathogens of international concern.**		
**Conduct timely and accurate disease reporting in accordance with WHO requirements.**		
**Collaborate with local public health officials and the healthcare delivery system, initiate active surveillance to identify additional cases during an epidemiologic investigation.**		
**Manage a national laboratory system and effective modern point-of-care and laboratory-based diagnostics.**		
**Participate in multinational epidemiologic studies.**		

	Importance of a job (A)	Ability to perform (B)
**Have the biological, clinical, and epidemiological knowledge needed to characterize (potentially novel) pathogens and other agents responsible for an outbreak disease.**		
**Integrate and interpret information from a variety of local, national, and international sources regarding contaminants in air, soil, and water.**		

Policy development, adaptation, and implementation. Workforce groups include but are not limited to: health officials at the ministry level, public health epidemiologists, environmental sciences, and public health emergency response managers, national focal points for preparedness and response, public health legal advisors, and health care infection control experts.

Please rate the following activities according to the following criteria.

- (A) How important is this activity to the successful performance of your job? not at all important = 1; very important = 7

- (B) How well do you consider that are you able to perform the activity? not at all = 1; very proficient = 7

*

Only answer this question if the following conditions are met:
Answer was at question ' [G04Q23]' ($(document).on('ready pjax:scriptcomplete',function(){ // Define some text strings var originalModalText = "One or more Required Questions have not been answered. You cannot proceed until these have been completed."; var newModalText = "Please notice you haven't answered this question. Still, you can continue without answering."; // Modify the modal text var modalBody = $('.modal-body p').filter(function() { return $.trim($(this).text()) == originalModalText; }); if(modalBody.length > 0) { modalBody.text(newModalText); } }); The following training needs assessment will be done based on the expertise categories below. Please check one (or more) category that is most suitable for your expertise.)

	Importance of a job (A)	Ability to perform (B)
**Work with epidemiologists, microbiologists, environmental sciences and others to continuously evaluate evidence on patient treatment and infection control.**		

	Importance of a job (A)	Ability to perform (B)
**Regularly assess and, as needed, clarify existing policies and/or recommend/advocate measures and communicate them to health officials at the ministry level, border control officials, and others.**	☐	☐
**Share relevant information with healthcare, infection control, and patient transport experts, and solicit their feedback.**	☐	☐
**Seek and receive advice from public health professionals in making border control decisions and reflect to the public how and why these decisions have been made.**	☐	☐
**Be able to use data products from epidemiologists in providing advice in the development of trade and travel restrictions as tools of population-based disease control.**	☐	☐
**Communicate the necessity of policies calling for personal protective measures to mitigate personal risks for the public health professionals.**	☐	☐
**Aid the transfer of medical and related professionals across borders and facilities through standardized job descriptions of personnel in clinical settings.**	☐	☐
**Provide healthcare workers with clinical guidelines for emerging infections from abroad, especially those that may be carried by travelers and the severely contagious.**	☐	☐
**Before the response operation, ensure regular assessments of legal frameworks and propose/advocate measures to address gaps.**	☐	☐

	Importance of a job (A)	Ability to perform (B)
**Before the response operation, assess if the implementation of strategies, plans, and action plans requires any changes in these plans and strategies.**		
**Before the response operation, identify which triggers will require key decisions during outbreak response (keeping in mind that triggers may need to be modified to fit specific situations).**		
**Review the evidence on current or impending outbreaks; propose and advocate adaptations to policies as needed.**		
**Communicate policy/guidelines, weigh benefits and costs, understand concerns about implementation, and adapt policies related to border control.**		
**Continuously evaluate evidence on threats; communicate if border control policies need to be adapted.**		
**Share information with response managers and health officials at the ministry level to support decisions about appropriate countermeasures.**		
**Before response activities are taken, regularly review, test, and update the standard operating procedures and ensure that a multi-unit task force is available for the coordination and integration of relevant sectors during response operations.**		
**Before the response operation, ensure the adequacy of plans for financing and credentialing of staff during emergency situations.**		

Health services: Workforce groups include but are not limited to: national Focal Point for preparedness, public health emergency response managers, health officials at the ministry level, public-sector healthcare delivery system managers, liaison workers for non-governmental hospitals, health professionals, procurement officials, manager members of medical products and technology, local public health institutions, healthcare infection control experts

Please rate the following activities according to the following criteria.

- (A) How important is this activity to the successful performance of your job? not at all important = 1; very important = 7

- (B) How well do you consider that are you able to perform the activity? not at all = 1; very proficient = 7

*

Only answer this question if the following conditions are met:
Answer was at question ' [G04Q23]' ($(document).on('ready pjax:scriptcomplete',function(){ // Define some text strings var originalModalText = "One or more Required Questions have not been answered. You cannot proceed until these have been completed."; var newModalText = "Please notice you haven't answered this question. Still, you can continue without answering."; // Modify the modal text var modalBody = $('.modal-body p').filter(function() { return $.trim($(this).text()) == originalModalText; }); if(modalBody.length > 0) { modalBody.text(newModalText); } }); The following training needs assessment will be done based on the expertise categories below. Please check one (or more) category that is most suitable for your expertise.)

	**Importance of a job (A)**	**Ability to perform (B)**
**Before an event, plan for the storage and stockpiling of vaccines and prepare for medical and non-medical countermeasures.**		

	Importance of a job (A)	Ability to perform (B)
**Draw upon the work of surveillance networks to identify potential events that may indicate the need for the implementation of preventative services plans.**		
**Ensure that plans are in place for mass vaccinations and mass prophylactic medication distribution.**		
**Coordinate vaccination plans and criteria for vaccination target groups to ensure consistency of practices.**		
**Facilitate the approval of vaccines through streamlined processes where available.**		
**Address antimicrobial stewardship activities.**		
**Prior to an event, work in tandem with clinicians to develop medical surge plans for various threats.**		
**Ensure that plans across the continuum of care have been communicated to the clinical staff to effectively manage surge needs.**		
**Plan for combining resources at national and local levels (e.g. cross-border sharing of clinicians if a hospital reaches capacity).**		
**Establish processes for staffing related surge issues including credentialing, paying staff, channels of authority, extended crisis interventions, and livelihood protection at home.**		

	Importance of a job (A)	Ability to perform (B)
**Establish reliable systems for disseminating case definitions to standardize both the diagnosis and the reporting of case numbers (e.g. confirmed, suspected, probable, or possible cases).**		
**Assess laboratory capacity on an ongoing basis and train public health scientists in rapid testing procedures to ensure adequate surge capacity.**		
**Create a hospital-based unit for critical, contagious patients at select facilities known to medical evacuation teams.**		
**Work with health personnel to identify the best medical countermeasures based on risk and threat; relay the results of these conversations.**		
**Ensure flexible policies and procurement strategies including how to allocate resources in the event of a shortage.**		
**Ensure there are adequate levels of human resources (e.g. experts) and laboratory capacity available.**		
**Use standardized approaches to engage with all personnel who may serve in field operations on the use of PPE.**		
**Before a response operation, relay to healthcare workers the importance of their role in public health emergencies and support their personal preparedness and that of their families.**		
**Establish ways to procure PPE for medical professionals and emergency responders.**		

	Importance of a job (A)	Ability to perform (B)
**Plan for the demobilization and recovery of the healthcare workforce after a response operation.**		

Coordination and communication (within the public health emergency preparedness system): Workforce groups inlcude but are not limited to: National focal point for preparedness, health officials at the ministry level, national public health agency leaders public health, public health emergency preparedness planners, civil protection agency for preparedness, public-sector healthcare delivery system managers, liaison with nongovernmental hospitals and health professionals.

Please rate the following activities according to the following criteria.

- (A) How important is this activity to the successful performance of your job? not at all important = 1; very important = 7

- (B) How well do you consider that are you able to perform the activity? not at all = 1; very proficient = 7

*

Only answer this question if the following conditions are met:
Answer was at question ' [G04Q23]' ($(document).on('ready pjax:scriptcomplete',function(){ // Define some text strings var originalModalText = "One or more Required Questions have not been answered. You cannot proceed until these have been completed."; var newModalText = "Please notice you haven't answered this question. Still, you can continue without answering."; // Modify the modal text var modalBody = $('.modal-body p').filter(function() { return $.trim($(this).text()) == originalModalText; }); if(modalBody.length > 0) { modalBody.text(newModalText); } }); The following training needs assessment will be done based on the expertise categories below. Please check one (or more) category that is most suitable for your expertise.)

	**Importance of a job (A)**	**Ability to perform (B)**
**Continuously create and update an incident management plan that adapts existing policies to the situation at hand.**		

	Importance of a job (A)	Ability to perform (B)
**Continuously inform public health emergency response managers about the threat so that the incident management plan can be updated.**		
**During the response operation, anticipate resource needs and communicate them to relevant decision makers.**		
**Before the response operation, practice and test the ability to make decisions under uncertainty.**		
**Participate in the implementation of plans which ensure the continuity of operations.**		
**Communicate with political decision makers to mobilize needed resources, communicate current knowledge and uncertainties, and solicit guidance.**		
**Before the response operation, identify key assumptions behind plans, identify untenable assumptions, and advocate changes as needed.**		
**Develop protocols and test/exercise processes for health emergency operations and their activation.**		
**Before the response operation, establish rapid communication channels within national disease surveillance and healthcare professionals.**		
**Before the response operation, establish trust with healthcare providers through feedback loops and two-way communication.**		
**For incident communication, draw on clinical personnel trained in risk communication or people involved in the incident, such as doctors or other clinicians.**		

	Importance of a job (A)	Ability to perform (B)
**Provide training; include healthcare providers in drills and exercises to test communication lines and avoid communication problems.**		
**Before the response operation, ensure that key partners are familiar with applicable laws, key roles, resources, information needs, and planning assumptions.**		
**Before the response operation, ensure adequate preparations for implementing health screening at borders; also ensure that response measures to a public health emergency can be taken right at the point of entry.**		
**Advocate the development of plans for joint task forces or other entities which can share information across disciplines.**		
**Advocate regular multi-discipline exercises to improve communication with staff and partners.**		
**Before the response operation, review mutual aid agreements (where relevant), identify gaps, and propose/advocate solutions to address gaps.**		
**Before the response, train staff members in confidentiality policies, chains of evidence, and security issues relating to the exchange of information between partner organizations.**		
**Identify key partners and develop a common understanding of roles, resources, planning assumptions, risks/vulnerabilities, and information that should be shared during response operations.**		

	Importance of a job (A)	Ability to perform (B)
**Develop strategies to communicate with professionals who have different skills and knowledge levels; develop strategies to communicate with partner organizations to ensure a coordinated response.**		
**Advocate regular multi-country exercises to improve the ability to communicate with partners.**		
**Assess the quality of the microbiology networks.**		
**Assess the adequacy of mutual aid mechanisms and multi-disciplinary taskforces.**		

Emergency risk communication (with the public). Workforce group includes but are not limited to: health officials at the ministry level, national public health agency leaders, risk communicators, civil society leadership, and professional journalists.

Please rate the following activities according to the following criteria.

- (A) How important is this activity to the successful performance of your job? not at all important = 1; very important = 7

- (B) How well do you consider that are you able to perform the activity? not at all = 1; very proficient = 7

*

Only answer this question if the following conditions are met:
Answer was at question ' [G04Q23]' ($(document).on('ready pjax:scriptcomplete',function(){ // Define some text strings var originalModalText = "One or more Required Questions have not been answered. You cannot proceed until these have been completed."; var newModalText = "Please notice you haven't answered this question. Still, you can continue without answering."; // Modify the modal text var modalBody = $('.modal-body p').filter(function() { return $.trim($(this).text()) == originalModalText; }); if(modalBody.length > 0) { modalBody.text(newModalText); } }); The following training needs assessment will be done based on the expertise categories below. Please check one (or more) category that is most suitable for your expertise.)

	Importance of a job (A)	Ability to perform (B)
**Address cultural and societal barriers in the cognitive processing and compliance with recommended behaviors.**		
**Use most appropriate content and trusted channels of communication across population groups.**		
**Identify strategies to overcome linguistic barriers, e.g. request local assistance.**		

	Importance of a job (A)	Ability to perform (B)
**Identify data gathering mechanisms to understand and monitor the informational needs of the population.**		
**Prevent and counter misinformation.**		
**Proactively address the needs of the news media and the general public.**		
**Integrate the results of the risk-assessment process in the messages.**		
**Manage and assess situational information received by the organization.**		
**Anticipate questions from the public and develop appropriate answers.**		
**Understand and implement the principles of risk communication.**		
**Identify strategies to facilitate the release of information (i.e. review outgoing messages in a timely manner).**		
**Understand laws and regulations related to emergency risk communication.**		
**Provide information to the public on the roles and responsibilities of the various organizations involved in the response operation; try to understand the public's perception of the emergency.**		

	Importance of a job (A)	Ability to perform (B)
**Identify strategies to engage with government leaders in order to integrate government priorities and community interests; address concerns that surface during the emergency response.**		
**Identify communication mechanisms that are trusted by the public, partners, and community influencers.**		
**Empower the public to participate in open discussions; involve the public in decisions relevant to public health threats.**		

Perceived Epidemic and Pandemic Preparedness Barriers and Potential Training Topics

In your opinion and based on your experience, what are the main barriers to epidemic and pandemic preparedness in the setting you are in. Please list them in order of importance.

In your opinion and based on your experience, what are the main barriers to attending epidemic/pandemic preparedness training(s) in the setting you are in? Please list them in order of importance.

Please specify the epidemic and pandemic preparedness capacities/skills in which you or your team would like to receive further training or instruction. Please list them in order of importance.

Thank you for your participation

The training needs assessment (section 4) was retrieved from: European Centre for Disease Prevention and Control. Public health emergency preparedness – Core competencies for EU Member States. Stockholm: ECDC; 2017.

Submit your survey.
Thank you for completing this survey.
