# Supplementary material for: Developing context-specific competencies for epidemic and pandemic preparedness in the MENA region: a training needs assessment and Delphi approach
Source: Front Public Health. 2026 Apr 20;14:1778190. doi: 10.3389/fpubh.2026.1778190 (PMC13136252; doi:10.3389/fpubh.2026.1778190)
Supplement: Supplementary file 3 [file Data_Sheet_3.PDF]

Detection and Assessment (n=48)			
Abbreviated Activity Name	Importance to Job	Ability to perform	Difference
*C1***	6.1	5.2	0.91
*C2***	6.1	5.5	0.65
*C3**	6.0	5.2	0.74
*C4**	6.2	5.1	1.05
*C5**	6.0	5.3	0.67
*C6**	6.0	5.3	0.71
*C7***	6.1	5.3	0.86
*C8**	5.8	5.0	0.85
*C9***	5.9	5.0	0.97
C10**	6.0	5.0	1.05
*C11**	6.1	5.5	0.57
*C12**	6.1	5.6	0.50
*C13***	5.9	4.9	0.95
*C14***	5.9	5.1	0.80
*C15***	5.9	5.0	0.93
C16**	5.9	4.5	1.36
C17**	5.9	4.6	1.34
C18**	6.0	4.8	1.22
C19**	5.9	4.5	1.39
*C20**	5.9	5.1	0.80
*C21***	5.9	5.0	0.87
C22**	5.6	4.1	1.48
*C23**	5.6	5.1	0.50
*C24***	5.9	4.7	1.19
*C25***	5.4	4.4	0.94

Policy Development (n = 51)			
Abbreviated Activity Name	Importance to Job	Ability to perform	Difference
*C1****	6.4	5.5	0.82
*C2***	6.2	5.5	0.70
*C3**	6.2	5.3	0.88
*C4**	5.7	5.0	0.69
*C5****	6.1	4.6	1.46
*C6**	6.3	5.7	0.61
*C7***	5.2	4.1	1.16
*C8**	6.0	5.4	0.62
*C9****	5.8	4.8	1.00
*C10**	6.0	5.2	0.80
*C11****	6.0	5.1	0.95
*C12****	6.2	5.5	0.76
*C13***	5.8	5.0	0.87
*C14***	5.7	4.8	0.92
*C15**	6.1	5.6	0.51
*C16****	6.2	5.1	1.10
*C17***	5.7	4.6	1.07

Coordination and Communication (n=51)			
Abbreviated Activity Name	Importance to Job	Ability to perform	Difference
C1*	6.3	5.1	1.25
C2*	6.1	4.9	1.14
C3*	6.4	5.2	1.19
C4**	6.0	4.7	1.28
C5*	6.2	5.2	0.93
C6*	6.1	5.3	0.84
C7*	6.0	5.0	1.02
C8*	6.2	5.0	1.21
C9**	6.1	5.2	0.88
C10*	6.2	5.3	0.83
C11*	5.9	4.9	0.93
C12*	6.0	5.1	0.88
C13*	5.8	4.8	0.98
C14*	5.9	4.7	1.24
C15*	6.2	5.2	0.95
C16	6.1	5.4	0.67
C17*	5.8	5.0	0.86
C18*	5.8	4.9	0.86
C19*	6.2	5.1	1.05
C20*	6.1	5.2	0.88
C21*	5.7	4.5	1.19
C22*	5.5	4.0	1.55
C23*	5.7	4.5	1.14

Emergency Risk Communication (n=37)			
Abbreviated Activity Name	Importance to Job	Ability to perform	Difference
*C1***	5.9	5.2	0.72
*C2***	6.1	5.1	0.93
*C3**	5.9	4.9	1.00
*C4***	6.0	4.9	1.10
*C5****	6.1	5.0	1.14
*C6**	5.9	5.4	0.55
*C7***	6.1	5.3	0.76
*C8***	5.9	5.1	0.82
*C9**	5.8	5.4	0.45
*C10***	6.2	5.4	0.86
*C11**	6.0	5.4	0.59
*C12**	6.0	5.1	0.93
*C13**	5.8	5.0	0.76
*C14**	5.9	5.2	0.76
*C15**	6.0	5.3	0.69
*C16**	6.0	4.9	1.10

Health Services (n=45)			
Abbreviated Activity Name	Importance to Job	Ability to perform	Difference
*C1****	5.9	4.6	1.34
*C2****	5.9	4.9	1.04
*C3****	6.1	4.7	1.47
*C4****	6.0	5.0	1.02
*C5****	5.9	4.8	1.18
*C6***	6.1	5.1	0.96
*C7****	6.1	5.1	1.03
*C8****	6.1	5.0	1.10
*C9****	6.1	4.8	1.34
*C10****	5.9	4.7	1.19
*C11***	6.2	5.6	0.63
*C12****	6.0	4.5	1.51
*C13****	6.2	5.1	1.14
*C14****	5.9	5.1	0.82
*C15****	5.9	4.7	1.17
*C16****	6.0	4.7	1.23
*C17***	6.1	5.3	0.77
*C18****	6.0	4.9	1.11
*C19***	6.2	5.1	1.12
*C20****	5.7	4.4	1.34
